# Biomaterials to Prevent Post-Operative Adhesion

**DOI:** 10.3390/ma13143056

**Published:** 2020-07-08

**Authors:** Heekyung Park, Seungho Baek, Hyun Kang, Donghyun Lee

**Affiliations:** 1 Department of Biomedical Engineering, School of Integrative Engineering, Chung-Ang University, 221 Heukseok-Dong, Dongjak-Gu, Seoul 06974, Korea; eldorado9228@naver.com (H.P.); snb3712@gmail.com (S.B.); 2 Department of Anesthesiology and Pain Medicine, Chung-Ang University College of Medicine and Graduate School of Medicine, Seoul 06973, Korea

**Keywords:** anti-adhesion barrier, biomaterials, open surgery, post-operative adhesion

## Abstract

Surgery is performed to treat various diseases. During the process, the surgical site is healed through self-healing after surgery. Post-operative or tissue adhesion caused by unnecessary contact with the surgical site occurs during the normal healing process. In addition, it has been frequently found in patients who have undergone surgery, and severe adhesion can cause chronic pain and various complications. Therefore, anti-adhesion barriers have been developed using multiple biomaterials to prevent post-operative adhesion. Typically, anti-adhesion barriers are manufactured and sold in numerous forms, such as gels, solutions, and films, but there are no products that can completely prevent post-operative adhesion. These products are generally applied over the surgical site to physically block adhesion to other sites (organs). Many studies have recently been conducted to increase the anti-adhesion effects through various strategies. This article reviews recent research trends in anti-adhesion barriers.

## 1. Introduction

**Figure 1:**
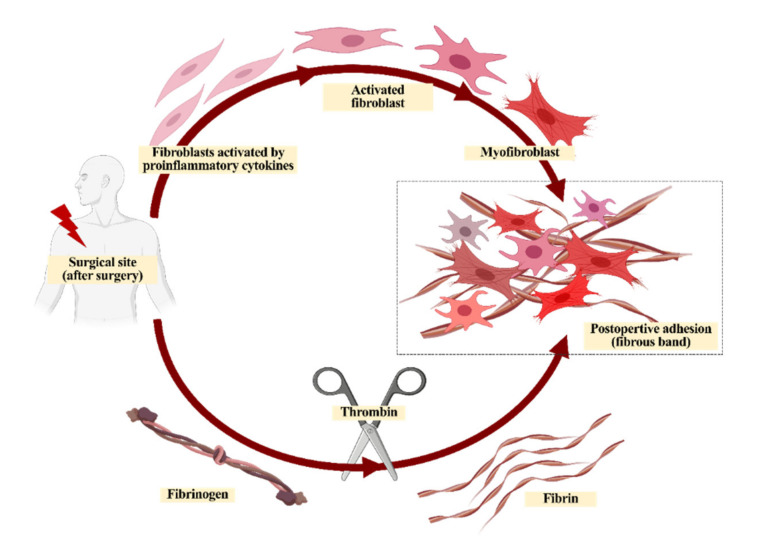
A simple schematic of the post-operative adhesion process.

Many surgeries are performed to treat a variety of diseases. The surgical site can be a small to large wound, depending on the severity of the disease [1]. When the vascular tissue around the surgical site is damaged during surgery, vascular endothelial cells, fibroblasts, and myofibroblasts grow in response to various hormones and cytokines in the blood, generating a fibrous band while entangling tissues for collagen repair [2,3,4,5,6] (Figure 1). Abdominal adhesion occurs at the surgical site due to contact with other sites during healing [7] and occurs in more than 90% of patients who undergo abdominal surgery.

**Figure 2:**
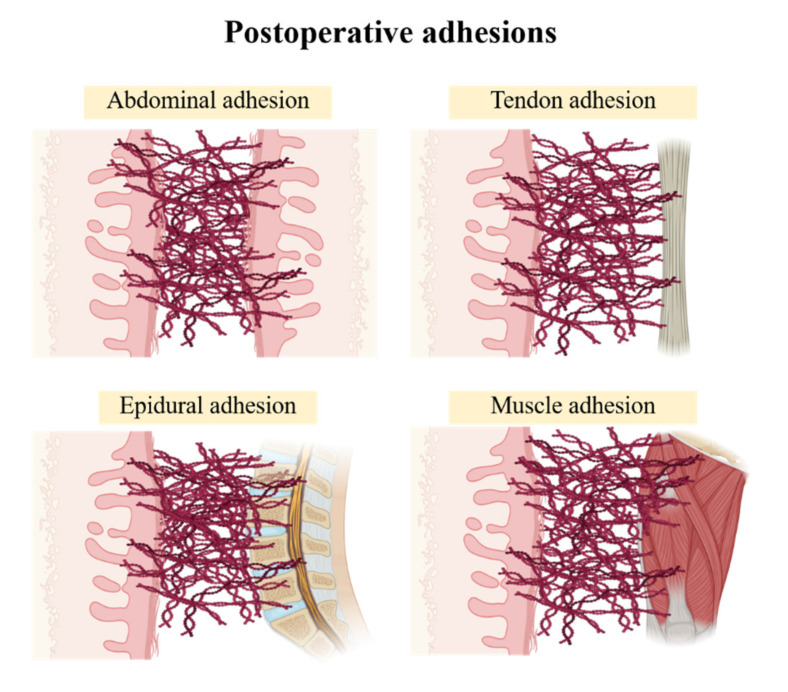
Various types of post-operative adhesions.

Post-operative adhesions are composed of fibrous scar tissue that connects the surgical site to the abdomen or other organs [8] (Figure 2). These can cause serious complications, such as abdominal pain, pelvic pain, infertility, and intestinal obstruction [9,10,11,12,13]. Additionally, postsurgical adhesion to the vertebral disks can cause paraplegia and severe pain, leading to fatal problems [14,15,16,17]. Many medical staff members and researchers are seeking ways to prevent tissue adhesion after open surgery through the development of various surgical methods, but it is difficult to completely prevent post-operative adhesion without the use of anti-adhesion barriers [18,19,20,21,22]. In general, the main target in the prevention of postoperative adhesion is to block or minimize the tissue ingrowth into anti-adhesion barrier materials, resulting in minimal connections between the surgical sites and other parts of the organs [2].

Various anti-adhesion barriers have been evaluated [14,23]. Anti-adhesion barriers generally block contact between the surgical site and other sites to inhibit fibrous band formation [2,24]. These products are classified into three forms: gels, solutions, and films [25]. Functional anti-adhesion barriers have recently been developed by loading drugs that prevent adhesion and have been used to treat surgical areas to prevent physically unnecessary adhesion [8,26] (Table 1).

Many commercial medical products have been developed to prevent post-operative adhesion [43]. Such medical products are manufactured using biomaterials, and most are nontoxic and biodegradable in the body [44]. The anti-adhesion barriers that have been commercialized thus far are summarized in Table 2. This review article also presents biomaterials that can be developed into anti-adhesion barriers and cites various studies on anti-adhesion barriers. Finally, this review article presents the current trends in anti-adhesion barrier research and discusses future research directions.

## 2. Polymers as Materials for Anti-Adhesion Barriers

Anti-adhesion barriers are usually developed using biomaterials. Their disadvantages are thus related to the biomaterials used [70]. Several studies have applied various biomaterials and technologies to establish anti-adhesion barriers [26,27,37,38,71,72]. These anti-adhesion biomaterials have excellent biocompatibility and biodegradability [73,74] and can be classified as either natural or synthetic polymers.

### 2.1. Natural Polymers

**Figure 3:**
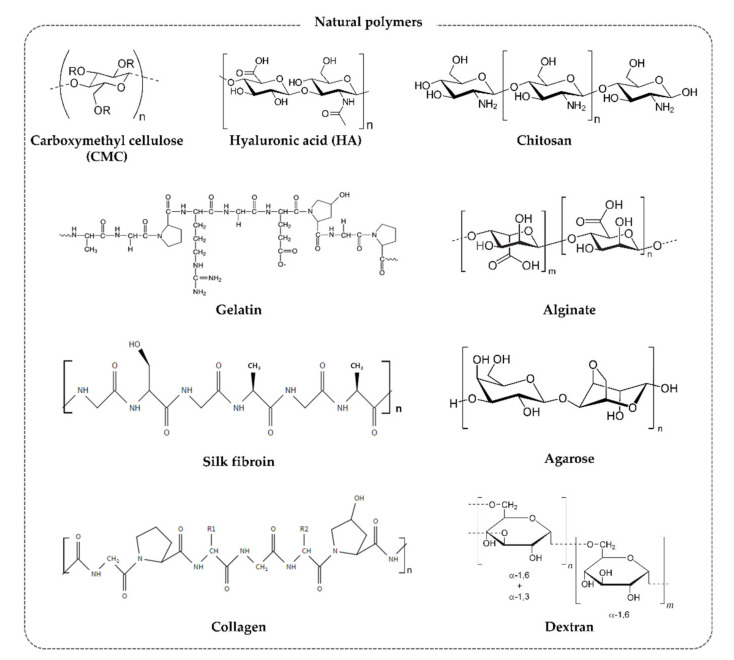
Structures of natural polymers (the image of the gelatin structure was adopted with permission [80]).

Natural polymers are derived from natural materials and animals (including humans) and exhibit excellent biocompatibility [75]. Gelatin and polysaccharide-based polymers such as alginate and hyaluronic acid (HA) are commonly used as anti-adhesion barriers [72,76,77,78]. The disadvantage of natural polymers is that it is challenging to maintain them in the body for an extended period due to their poor physical properties. However, they are easy to process, and FDA approval can be obtained relatively easily [79]. As such, natural polymer anti-adhesion barriers have been widely developed (Figure 3).

#### 2.1.1. Carboxymethyl Cellulose (CMC)

Carboxymethyl cellulose (CMC) is synthesized by an alkali-catalyzed reaction between cellulose and chloroacetic acid [81]. It has the chemical formula C_6_H_7_O_2_(OR_1_)(OR_2_)(OR_3_), where R_1_, R_2_, and R_3_ are H or C_8_H_16_NaO_8_. It is widely used in biomedical engineering, tissue engineering, and drug delivery systems [82,83,84,85]. Generally, cellulose is insoluble in water, but CMC exists as water-soluble salts. It has good biocompatibility and biodegradability and can be manufactured and applied as a hydrogel or film [26,86]. As an anti-adhesion barrier, seprafilm^®^ and intercoat are commercialized using CMC [43]. Most CMC-based anti-adhesion barriers have been developed into medical products that are absorbed into the body within a few days. These CMC-based barriers are manufactured by electrospinning and solvent casting methods, and anti-adhesion barriers that are more effective than the current commercial products are being actively developed by loading these barriers with drugs [26,87].

#### 2.1.2. Hyaluronic Acid (HA)

HA is a natural linear polymer consisting of repeating units of N-acetyl glucosamine and gluconic acid [88] and has the formula (C_14_H_21_NO_11_)_n_. It has been widely used in cosmetics and pharmaceuticals [89]. HA has a good swelling ability and biocompatibility [90,91]. In addition, using HA is advantageous because its mechanical properties can be controlled through crosslinking reactions [92]. However, HA is absorbed in the body within 3 days and is then rapidly decomposed, and therefore, it is difficult to use HA as a single material in anti-adhesion barriers. Guardix-sol^®^, a currently commercialized product, is made by blending HA and CMC and can exist in the body for more than two weeks. This longevity and its anti-adhesion properties have led to Guardix-sol^®^ being sold as an anti-adhesion barrier for use at surgical sites [49].

#### 2.1.3. Chitosan

Chitosan is a natural amine-containing polysaccharide with various useful biological properties, including excellent biocompatibility, biodegradation, nontoxicity, hemostatic activity, antibacterial activity, and free radical-scavenging activity [93,94]. Chitosan also has a longer decomposition time in the body than other biomaterials, resulting in its application as an anti-adhesion barrier [95,96,97]. However, chitosan-derived anti-adhesion barriers can be dangerous to patients allergic to chitin, so they are not applicable for those cases.

#### 2.1.4. Gelatin

Gelatin is a biomaterial that can be obtained from the extracellular matrix (ECM) layers of animals (including humans) and has excellent biocompatibility [98]. Therefore, it is widely applied to tissue engineering and medical products. Crosslinking (by agents such as glutaraldehyde (GTA), carbodiimides, and genipin) can be performed to control its mechanical properties [99]. However, unreacted crosslinking agents remaining after crosslinking reactions can be toxic, hampering the use of gelatin-based materials for medical products [100]. Recently, nanotechnology has been applied to develop effective chemical anti-adhesion barriers using gelatin or drug loading [101,102,103].

#### 2.1.5. Alginate

Alginate is abundant in seaweed, is a polysaccharide block copolymer in which α-L-guluronic acid and β-D-mannuronic acid are repeated [104], and has the chemical formula (C_6_H_8_O_6_)_n_. Linear alginate is soluble in water and gelated by interactions with ions such as Ca^2+^ and Ba^2+^ [105]. These crosslinking reactions are nontoxic and are advantageous because they can be used to control the mechanical properties of the polymer [104]. Nanofiber-type anti-adhesion barriers have recently been developed using electrospinning, and furthermore, effective anti-adhesion barriers have been developed by loading materials with drugs with anti-adhesion effects [26,106,107]. 

### 2.2. Synthetic Polymers

**Figure 4:**
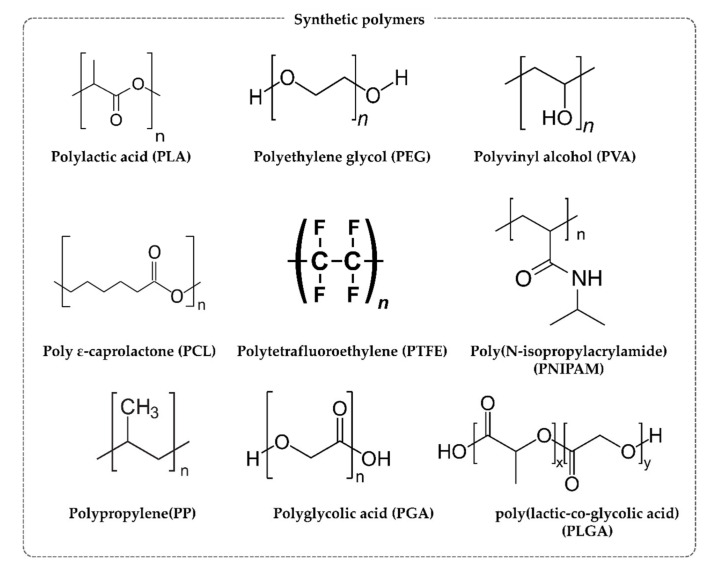
Structures of synthetic polymers.

Synthetic polymers that can be applied in the body are generally biocompatible and should be biodegradable [108]. In addition, the byproducts of decomposition should not cause complications and be nontoxic. Synthetic polymers biodegrade more slowly than natural polymers [109]. Anti-adhesion barriers developed with synthetic polymers can, therefore, be expected to exert longer-lasting anti-adhesion effects than those produced with natural polymers [110]. Synthetic polymers are composed of repeating units, allowing the easy control of properties such as the molecular weight, and they have excellent mechanical properties. In addition, since they are hydrolyzed in the body or decomposed by enzymes, they are particularly useful polymers as bioimplants. Typical biodegradable synthetic polymers, the polyester-based polymers, include polylactic acid (PLA), polyglycolic acid (PGA), and poly ε-caprolactone (PCL) (Figure 4).

#### 2.2.1. Polylactic Acid (PLA)

Polylactic acid (PLA) is synthesized from the in vivo metabolite’s lactic acid or lactide through a ring-opening reaction by a chemical catalyst or an enzyme. The chemical formula of PLA is [–C(CH_3_)HC(=O)O–]_n_. As a monomer, lactic acid has D and L optical isomers, and the properties of PLA can be varied by changing the ratio between the D form and L form [111]. PLA has a high melting point and excellent strength, but poor processability, poor flexibility, and high cost [38]. A method for producing lactic acid by fermenting corn was recently developed and has attracted attention due to its reduced manufacturing cost. Additionally, since PLA is biodegradable, it has been used in the development of medical materials, such as medical sutures and bioimplants [112].

#### 2.2.2. Polyvinyl Alcohol (PVA)

Polyvinyl alcohol (PVA) has the formula [CH_2_CH(OH)]_n_. PVA is a water-soluble, semi-crystalline polymer that has excellent thermal stability, physical properties, and biocompatibility and is inexpensive [113,114]. PVA has a strong oxygen barrier due to strong hydrogen bonds, but the presence of hydroxyl groups makes it very susceptible to moisture, causing swelling and dissolving. To address this issue, PVA is crosslinked and used as a hydrogel [112]. Dianhydride and dialdehyde are mainly used as the crosslinking agents, but a hydrogel can be obtained by using UV light or repeated freezing and thawing [115]. PVA is being studied for use not only in paints, coatings, and adhesives, but also in biomedical fields, such as in artificial cartilage, eye drops, and contact lenses. It is widely applied in the development of anti-adhesion barriers by being blended with other synthetic polymers and natural polymers [116,117].

#### 2.2.3. Poly ε-Caprolactone (PCL)

Poly ε-caprolactone (PCL) is relatively inexpensively produced compared to other aliphatic polyesters and is obtained by ring-opening polymerization from ε-caprolactone [118]. The structure is (C_6_H_10_O_2_)_n_. PCL has excellent mechanical properties, such as tensile strength, elongation, and impact strength, and a low melting point [119]. Additionally, PCL has the advantage of a good compatibility with other polymers and is biodegradable [120]. Since the degradability of PCL by lipase is affected when it has higher-order structures (represented by parameters such as the crystallinity), the biodegradation rate of PCL varies with the processing method [121]. PCL does not cause toxicity in vivo and is a biocompatible material, and it is thus used in medical sutures and as a drug release material for long-term wound closure [96]. This polymer is mainly prepared as a nanofibrous anti-adhesion barrier through electrospinning and can be combined with a natural polymer in a core-sheath structure to enhance the biocompatibility [122].

#### 2.2.4. Polyethylene Glycol (PEG)

**Figure 5:**
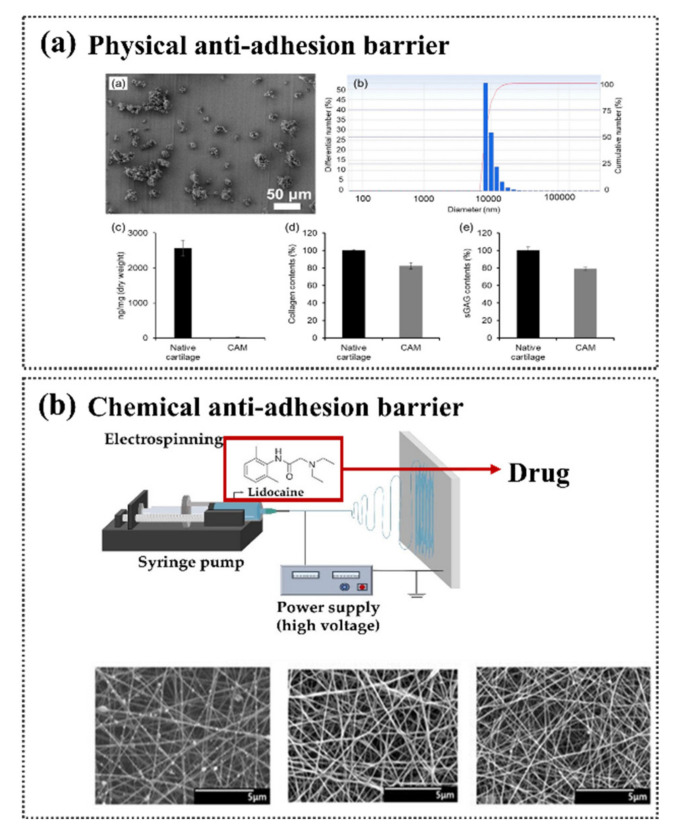
Physical and chemical anti-adhesion barrier: (**a**) Cross-linked cartilage acellular-matrix film [126]; (**b**) drug-loaded alginate/carboxymethyl cellulose (CMC)/polyethylene oxide (PEO) anti-adhesion film [26].

Polyethylene glycol (PEG) has the structure H–(OCH_2_CH_2_)_n_–OH. Generally, PEG is called polyethylene oxide (PEO) or polyoxymethylene (POE), depending on its molecular weight [123]. This polymer has good biocompatibility and is nontoxic [124]. In medical applications, irrigation with PEG is used for bowel preparation before surgery [125]. In pharmaceutical applications, PEG is used as an excipient [124]. Therefore, it has been applied as an anti-adhesion barrier in many studies. Currently, there are no products that can completely prevent tissue attachment at surgical sites. Therefore, many studies have been conducted to overcome the disadvantages of existing anti-adhesion barriers and to develop new effective anti-adhesion barriers. These barriers can be classified by their mode of operation as either a physical or chemical barrier [26,126] (Figure 5). The development of various manufacturing technologies has enabled the active study of new anti-adhesion barriers that can be expected to surpass the anti-adhesion barriers currently being commercialized [26].

## 3. Various Strategies of Anti-Adhesion 

### 3.1. Physical Barriers

As a first method to prevent adhesion, there is a method of blocking contact with the surgical site and surrounding tissue using an anti-adhesion barrier after surgery [127]. Second, there is a method of suppressing adhesion using drugs based on the mechanism of adhesion [128]. Finally, there is a method of minimizing adhesion by unnecessary tissue damage by using delicate surgery and minimally invasive surgery [129]. However, whilst minimally invasive surgical techniques can help to prevent adhesion to a level that minimizes or prevents exposure to foreign substances and tissue drying, there is the limitation that adhesion cannot be eliminated. Therefore, to effectively prevent and eliminate adhesion, the use of an anti-adhesion barrier is inevitable.

Physical barriers prevent unnecessary contact by blocking surgical sites from other organs [7], and most of the physical barrier medical products are decomposed and absorbed in the body [25]. In order to prevent adhesion during the wound healing period, which generally has a period of about 7 days, a physical barrier that can remain undissolved can more effectively prevent adhesion. Physical barriers are typically hydrogels or films.

Recently, studies have been conducted in which polymer mesh and hydrogel are combined to enhance the effectiveness of preventing post-operative adhesion (e.g., polypropylene mesh combined with hydrogel) [130,131].

#### 3.1.1. Hydrogels

Hydrogels have been studied since the 1960s. A hydrogel is a water-soluble polymer network structure connected in three dimensions by physical and chemical bonds [132]. Hydrogel materials contain large amounts of water and do not dissolve in aqueous environments. Various hydrogel characteristics depend on the polymer constituents, and hydrogels can be transformed into multiple forms due to their ease of processing [133,134].

Hydrogels mainly absorb water via hydrophilic functional groups such as hydroxyl (–OH), amine (–CNH_2_), amide (–CONH– and –CONH_2_), carboxyl (–COOH), and sulfone (–SO_3_H) groups and by capillary and osmotic pressures [135,136,137]. Hydrophilic and hydrophobic hydrogels maintain their three-dimensional forms without being dissolved in water because the dispersion force in the water and their cohesiveness are in equilibrium.

**Figure 6:**
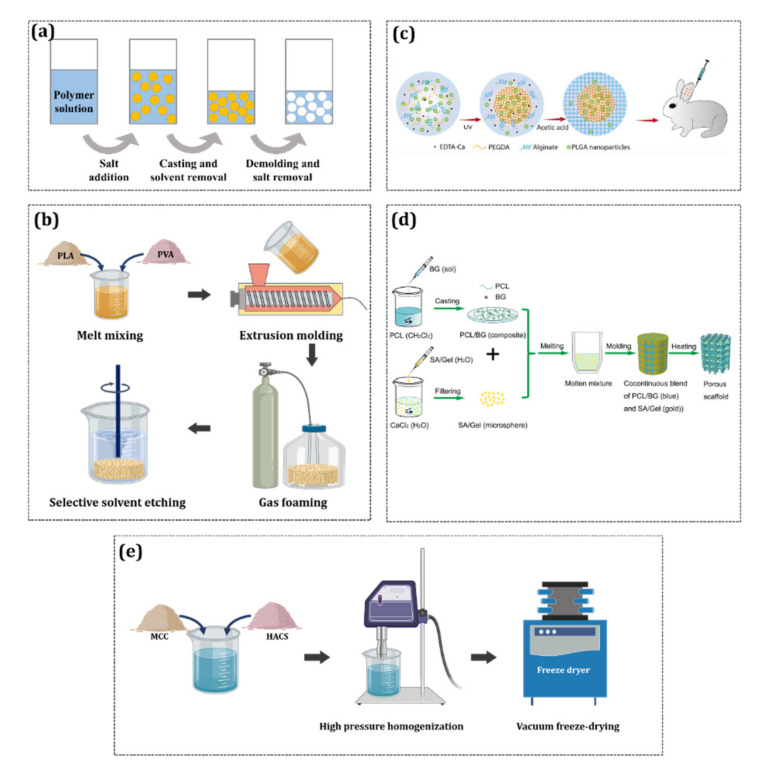
Hydrogel fabrication techniques: (**a**) Solvent-casting/particulate-leaching [138]; (**b**) gas foaming [139]; (**c**) phase separation [140]; (**d**) melt molding [141]; and (**e**) freeze drying [142] (images adopted with permission).

Hydrogels are made by techniques such as solvent casting/particulate leaching [138], gas foaming [139], phase separation [140], melt molding [141], and freeze-drying [142] (Figure 6). Hydrogels can be manufactured as films, coatings, nanoparticles, etc., and can act as semipermeable membranes through which fluid can flow in three dimensions. Additionally, hydrogels have been widely used in laboratories and clinical trials because they have high biocompatibility, which is due to their high similarity to the ECM when a large amount of water has been absorbed [143].

Hydrogels can be chemically or physically crosslinked [144]. Physical methods do not require a crosslinking agent and can produce hydrogels reversibly, but a nonuniform structure is formed. Strong physical interactions are formed in glassy nodules, lamellar microcrystals, and double and triple helices, while hydrogels are formed by weak physical forces, such as ionic bonds, hydrogen bonds, or the self-assembly forces in block copolymer micelles [145].

Recently, a thermosensitive hydrogel was developed to apply an anti-adhesion barrier, even in surgery, using the minimally invasive method. Thermosensitive anti-adhesion barriers have the advantage that they can be applied to surgical sites through injection because they are liquid at room temperature and gel at body temperature [27,32,146,147].

#### 3.1.2. Films

Nanofibrous films with a uniform pore size, high porosity, and good permeability can effectively prevent adhesion [148]. Typical techniques for making nanofibrous films are electrospinning, self-assembling peptide reactions, and phase separation [149,150,151]. Among them, electrospinning technology can form exceptionally long fibers and exhibits a high productivity in a short time [152].

Nanofibrous films obtained through electrospinning have a high specific surface area, porosity, aspect ratio, and flexibility. Additionally, the diameter can be easily adjusted based on various conditions. Electrospinning can be applied to multiple polymers, but toxic solvents are sometimes used, representing a disadvantage. However, electrospinning is the best method for commercially producing nanofiber films [153,154,155]. Nanofibrous films can be used in various applications, such as in stiffeners, high-efficiency filters, functional fibers, and munitions, and in other medical fields. In the medical field, nanofiber films are used in drug delivery systems, scaffolds for tissue engineering, and wound dressings [156,157,158].

**Figure 7:**
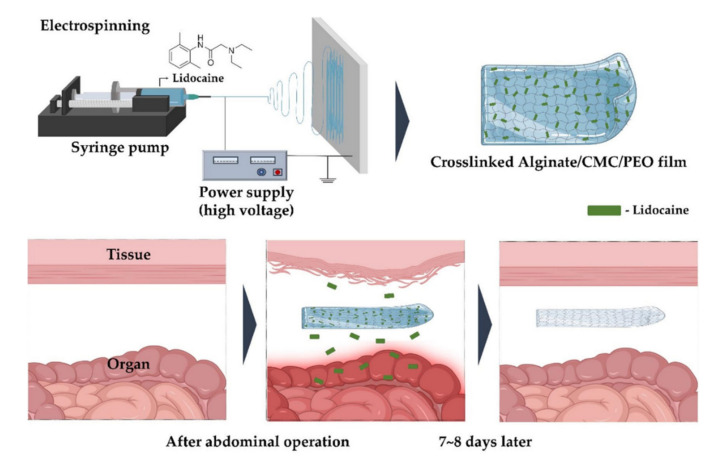
Fabrication of nanofibrous films by electrospinning [26].

In electrospinning, a polymer is radiated to a collector through a nozzle by an electrostatic force generated by a high voltage of kV or more and is stretched to a diameter of tens to hundreds of nanometers. When the charge generated on the surface of the polymer solution extruded from the nozzle becomes larger than the surface tension, a jet is created from the Taylor cone and is drawn into the microfiber through bending instabilities [159,160]. Nanofibers are influenced by material factors such as the concentration, structure, elasticity, conductivity, polarity, and surface tension, and mechanical elements such as the electric field strength, tip-to-collector distance, and flow rate [161,162] (Figure 7).

### 3.2. Chemical Barriers

**Figure 8:**
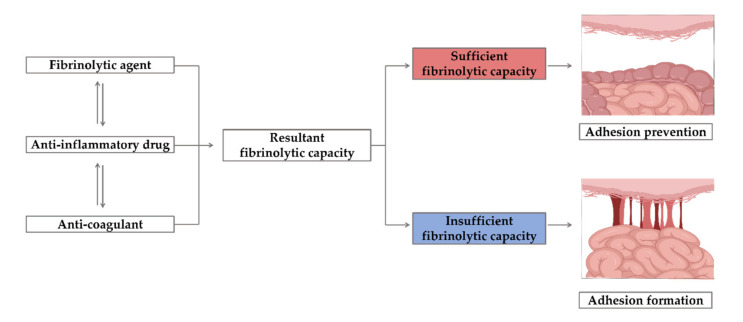
The effects of fibrinolytic agents, anti-inflammatory drugs, and anti-coagulants as anti-adhesion agents.

Research has been conducted to prevent adhesions by finding the factors contributing to adhesion based on the adhesion mechanism. Most anti-adhesion drugs interfere with the deposition of fibrin, but slow wound healing, in addition to having anti-adhesion effects [163,164,165]. Fibrin is a poorly soluble glycoprotein that causes blood clotting and is produced by the hydrolysis of fibrinogen by thrombin. Fibrin intertwines with blood cells to become a blood clot and hemostatic. It is decomposed through fibrinolysis. If this action does not occur sufficiently, the fibrin matrix forms an adhesion [166]. Three types of drugs are used to prevent adhesions. The first type is anti-inflammatory agents, which include steroids, nonsteroidal anti-inflammatory drugs (NSAIDs), vitamin E, and low-dose aspirin [167,168]. Second, the anticoagulants warfarin; coumarin; indirect thrombin inhibitors; and direct thrombin inhibitors, such as hirudin, bivalirudin, and argatroban, are also used [169,170]. Finally, the fibrinolytic agents streptokinase, urokinase, and tissue plasminogen activator (tPA) are used [44,171]. All three types of drugs ultimately lead to fibrinolytic capacity (Figure 8).

The physical barrier methods only act on a local site, but loading a drug in a material leads to the therapeutic expectation of a more effective anti-adhesion effect. However, there is no consensus on the anti-adhesion effects of drugs, and thus, there is no commercially available drug-loaded anti-adhesion barrier [163,172].

#### 3.2.1. Anti-Inflammatory Agents

Inflammation is a defense mechanism against tissue damage, injury, infectious agents, and autoimmune reactions and is an essential part of the autoimmune response. Inflammatory reactions have effects that include redness, edema, pain, and loss of function [173].

After surgery, blood flow increases at the site of injury, vascular permeability increases, and immune cell migration occurs. Activated inflammatory cells (neutrophils, eosinophils, mononuclear phagocytes, and macrophages), which are present at the site, secrete small molecules such as prostaglandins (PGs) and nitric oxide (NO) and cytokines such as interleukin (IL)-1β, IL-6, and tumor necrosis factor (TNF). In particular, a specific amount of the free-radical NO is required in the body for signaling, particularly for thermoregulation, vasodilation, and neuromodulation, and this molecule is produced by the enzymes endothelial nitric oxide synthase (eNOS), neuronal nitric oxide synthase (nNOS), and inducible nitric oxide synthase (iNOS). If excess NO is produced by iNOS, whose expression is induced during inflammation, it can cause hypotension due to shock, damage to nerve tissue, and tissue damage by inflammatory reactions. Therefore, when an anti-inflammatory drug is used, iNOS is suppressed, resulting in an anti-adhesion effect [174,175,176].

Studies have been conducted to prove the anti-adhesion effect of loading anti-inflammatory agents, e.g., NSAIDS such as ibuprofen [177], celecoxib [167], naproxen [178], and aspirin [179]. Research has also been conducted using natural anti-inflammatory agents, such as green tea extract [180] and Turkish galls extract [40].

#### 3.2.2. Anticoagulants

Blood coagulation is a biological reaction that occurs to minimize the loss of blood from damaged blood vessels and a biological defense mechanism that maintains the intrinsic function of blood through maintaining blood circulation. Maintaining blood circulation is possible through efficient control of the blood coagulation reaction system and complementary control of the thrombolysis reaction system [181,182].

When an atherosclerotic plaque in the blood vessel ruptures, coagulation factor VII is activated by an exposed tissue factor, and the coagulation system is activated. Thrombin converts fibrinogen to fibrin and activates several other coagulation factors to promote platelet aggregation [183,184]. Unfractionated heparin and low-molecular-weight heparin, which are actually used as anticoagulants, activate antithrombin III and indirectly inhibit thrombin’s action [170,185]. Hirudin directly inhibits thrombin’s action, and warfarin inhibits the production of blood coagulation factors [186].

Although studies have demonstrated anti-adhesive effects of anticoagulants such as hirudin [186], heparin [187,188], DMSO [189], and thrombolytic protein from cobra venom [190], some studies have shown that the effects are ambiguous [185]. Therefore, it seems that more research is needed to prove the anti-adhesion effects of anticoagulants.

#### 3.2.3. Fibrinolytic Agents

Fibrinolytic agents dissolve thrombi and inhibit the formation of excessive thrombi, thereby maintaining the openness of blood vessels. The most important factor is plasminogen, which is converted into plasmin by a plasminogen activator (PA) to dissolve fibrin and form a fibrin degradation product to achieve thrombolysis. Plasmin can dissolve fibrinogen and fibrin, but the reaction is local. There are two types of Pas: t-PA and u-PA. t-PA is mainly involved in fibrinolysis in circulating blood, and u-PA binds to receptors and increases the activity of plasminogen [191,192,193].

Studies have been conducted to prove that N-acetyl-L-cysteine [194], which upregulates peritoneal fibrinolytic activity or the fibrinolytic agent streptokinase [195], has anti-adhesive effects. However, fibrinolytic agent use can lead to bleeding complications, and streptokinase has the disadvantage of inducing severe hypotension symptoms due to allergic reactions [196].

## 4. Discussion

Postoperative adhesion is a surgical complication that has not been completely overcome until now. In addition, it has an incidence rate of 90% during recovery after surgery, and most postoperative adhesion incidences occur due to separation between the surgical site and the anti-adhesion barriers [7]. In severe cases, re-operation is often required to remove adhesions. However, re-operation causes additional wounds, which usually adds additional factors for adhesion [197,198]. In order to overcome such limitations, anti-adhesion barriers have been developed from different angles.

In our previous study, for example [107], we prepared alginate/PEO film as an anti-adhesion barrier through electrospinning. The focus of the study was the fact that alginate has negative charges due to the presence of carboxyl groups. Therefore, alginate/PEO film has strong negative charge characteristics, which was confirmed by cell attachment tests. Fibroblast cells were repelled and did not attach well to the films. Such characteristics are thought to help minimize cell migration into the scaffold layer, reducing adhesion at the surgical site. In animal studies, alginate/PEO film exhibited a significant reduction of postoperative adhesion compared to some commercial products [107]. Chang et al. fabricated chitosan/alginate mats via electrospinning [95]. Chitosan/alginate mats are known to exhibit hemostasis effects due to the presence of chitosan and reduction of protein adsorption due to the presence of alginate, which was confirmed by in vitro experiments and abdominal rat models [95]. Wang et al. prepared naproxen nanoparticle-loaded chitosan hydrogel [32]. Naproxen (Nap) is a non-steroidal anti-inflammatory drug (NSAID) prescribed in oral and suppository routes in clinics that are widely used to relieve pain and reduce inflammation. Naproxen nanoparticles were prepared by MPEG-PCL copolymers, which were then loaded into chitosan hydrogel. The structure was prepared by linking the chitosan hydrogel and naproxen nanoparticle with *β*- Glycerol phosphate disodium salt pentahydrate. As a result, naproxen nanoparticle-loaded chitosan hydrogel showed a similar postoperative adhesion prevention effect to commercial products and also displayed stable drug release behavior [32].

A handful of studies have also been conducted to discover more effective alternative anti-adhesion materials among those that are not as commonly used as others in traditional anti-adhesion studies. Silk, which consists of two main proteins (sericin and fibroin), is a natural polymer that can be emitted by spiders and silkworms [199]. One of the main proteins in silk, sericin, is known to trigger an immune response [200]. Several studies have been conducted to apply silk fibroin protein for the development of anti-adhesion barriers. Silk fibroin is known for its good biocompatibility, and the main primary structure consists of (Gly-Ser-Gly-Ala-Gly-Ala)_n_ amino acid sequences [200]. In addition, the high glycine content contributes to the tensile strength and rigid structure of silk fibroin [201]. Zhu et al. prepared novel silkworm pupa carboxymethyl chitosan-based structures for anti-adhesion effects, and evaluated them via a rat cecal abrasion model [202]. Vepari et al. controlled the anti-adhesion effect by changing the degree of hydrophobicity through PEGylation of the surface of the silk fibroin film [203]. These studies show that raw silk fibroin has potential to be used as a candidate material for producing an anti-adhesion effect.

Agarose is a polysaccharide, generally derived from red seaweed. It is a liner polymer that consists of repeating units of disaccharides: D-galactose and 3,6-anhydro-L-galactopyranose. Standard agarose derived from *Gelidium* dissolves well in water with a close to boiling temperature and gels at 34–38 °C. For decades, agarose has been widely used as scaffold matrix material in gel electrophoresis, but has not been actively used for anti-adhesion purposes [204,205]. However, Tang et al. prepared agarose/collagen (with a ratio of 2:1, 4:3, and 1:1) anti-adhesion sheets, and evaluated the effect of anti-adhesion through in vitro and in vivo experiments. The sheets were cross-linked using glutaraldehyde for mechanical strength, and were shown to exhibit good anti-adhesion effects compared to control groups (without sheets) [206]. Although agarose is widely used in the field of tissue engineering and regenerative medicine (due to its nontoxicity and good biocompatibility), it has to be stressed that glutaraldehyde, one of the representative agarose crosslinking agents, needs to be carefully administered, for the residues left after the crosslinking reaction can cause severe cytotoxicity [207].

Collagen, known to be the most abundant single protein in the animal kingdom, is a major insoluble fibrous protein of extracellular matrices and connective tissues. The amino acid sequences of collagen are commonly known as (Gly-Pro-X-Gly-X-Hyp), where X can be any amino acid except Gly, Pro, and Hyp. Therefore, collagen exhibits excellent biocompatibility and nontoxicity [208]. Cai et al. were able to enhance the mechanical strength of a chitosan/carboxymethyl cellulose/collagen composite membrane by a transglutaminase-catalyzed crosslinking reaction, and tested the anti-adhesion effect by in vitro and in vivo experiments [31]. Dabrowski et al. showed that COVA+^TM^ made of collagen could effectively prevent postoperative adhesions that may occur after peritoneal surgeries [60].

Dextran is one of the polysaccharides of polymers and consists of complex branched glucan. Hyskon^®^ solution, a commercialized product, is a drug employed for hysteroscopy and has been found to have anti-adhesion effects through the control of immune cells [59].

Gore^®^Preclude^®^ (an expanded polytetrafluoroethylene (ePTFE) membrane) is a medical product that consists of pure PTFE and is used for cardiac surgery [69]. PTFE is a linear polymer consisting of fluorine and carbon, and is known to be hydrophobic. ePTFE, which exhibits a unique mechanical property, can be obtained in the form of a microporous fibrous membrane structure through the expansion process at a high temperature and high pressure. ePTFE is used in a variety of medical accessories, including vascular graft, suture, and wound-care products [209]. Lladó et al. selected Gore^®^Preclude^®^ as an anti-adhesion barrier to prevent peridural fibrosis in patients with spinal surgery and confirmed the prevention of adhesion through clinical studies. In spinal surgeries, the permanent removal of adhesion is thought to be important and ePTFE was thus thought to be a good candidate material for the procedure [210]. However, ePTFE is a non-biodegradable polymer, and it is thus difficult to apply it for various surgeries as commonly as other candidate materials [211].

Polyglycolic acid (PGA) is a biodegradable, thermoplastic polyester. PGA is widely used in copolymer forms due to its instability during hydrolysis [212]. Poly (lactic-co-glycolic acid) (PLGA) is a copolymer of PGA and lactic acid, which is one of the most well-known biomaterials [213]. Poly lactic acid or polylactide (PLA) is a thermoplastic and bioplastic polymer, and is mostly obtained by condensation reactions of lactic acid with a loss of water. PLA has been confirmed to take a full 10 months to completely degrade in physiological environments [111]. Therefore, PGA and PLA are difficult to use as standalone materials for an anti-adhesion barrier. PLGA is a biodegradable, aliphatic polyester-based synthetic polymer with good biodegradability and biocompatibility [213]. Niu et al. prepared a PLGA/poly(lactide-co-caprolactone) (PLCA)/poly(L-phenylalanine-co-p-dioxanone (PDPA) film using an electrospinning and casting method and evaluated the performance of the bilayer film through an abdominal rabbit model [38]. Polypropylene (PP) is a thermoplastic polymer and is widely used in various research fields. It can be obtained through chain-growth polymerization from propylene monomers. In the medical field, propylene is often used as a material for permanent medical meshes [214]. Sezer et al. prepared an anti-adhesion layer using PP mesh [39]. They prepared a PP/PCL/oxidized regenerated cellulose (ORC) layer by electrospinning ORC and PCL on top of the PP mesh. The fabricated PP/PCL/oxidized regenerated cellulose (ORC) layer was evaluated by in vitro and animal studies and was shown to exhibit a good adhesion prevention effect [39].

Poly(*N*-isopropylacrylamide) (PNIPAm or PNIPAAm) is a biodegradable, temperature-responsive polymer. PNIPAm’s degree of hydrophilicity and hydrophobicity is commonly determined based on its lower critical solution temperature (LCST) [215]. Such a property is widely applied in drug delivery and tissue engineering studies, as well as in the development of temperature-sensitive injections for preventing postoperative adhesions [146].

Various biomaterials and manufacturing processes are being tested in the hope of developing more effective anti-adhesion barriers. However, there are still no completely effective products and/or modes of action that can totally replace traditional products in the field. From functional points of view, physical barriers seem to have reached their limits. However, most commercial products are based on physical barriers, and products that can be classified as chemical barriers are not easily found. This is because chemical barriers can exert unwanted additional effects, and do not just simply prevent postoperative adhesions. Therefore, they may cause higher risks of side effects compared to physical barriers. Therefore, multiple studies are being conducted to effectively combine the physical barriers and chemical barriers to control the pattern and the amount of drug release. It is necessary to understand the limitations of the candidate materials for anti-adhesion and to study various aspects of biomaterials that can match the needs of specific surgical procedures and sites.

## 5. Conclusions

In modern medicine, open surgery is performed to treat diseases. However, it remains challenging to completely prevent tissue adhesion after open surgery. The key to preventing post-operative adhesion is to minimize the tissue ingrowth into the scaffold materials, which can lead to the effective blocking of the tissue connection. In addition to the material properties, one of the most important factors to consider in designing anti-adhesion barriers is to let the barriers stably fix on the surgical site. One way that the factor can be controlled is by controlling the biodegradation properties of the anti-adhesion barriers. The anti-adhesion barriers are decomposed after a certain period of time in the body, resulting in a change in the fixation force on the surgical sites. Therefore, many researchers have sought to develop effective anti-adhesion barriers by mitigating the disadvantages of existing anti-adhesion barriers and maximizing their advantages by controlling the degradation behaviors of the barriers. In addition, it is challenging to achieve minimal tissue ingrowth and stable fixation on the surgical site at the same time. There have been studies conducted in an effort to enhance fixation by incorporating polymeric mesh structures in the scaffold layer of the anti-adhesion barriers, and this is another aspect that can be considered in the development of new barrier products in the future as a possible solution to achieve both of the above [130,131].

It is necessary to deepen not only our understanding of the properties of materials, but also the methods of manufacturing used for the fabrication of anti-adhesion barriers. This review presents the current research trends in the development of anti-adhesion barriers and provides information required for future studies. It can provide the basis for developing more advanced and more effective anti-adhesion barriers.

## Figures and Tables

**Table 1:** Anti-adhesion barriers that have been the subject of recent research.

Biomaterial	Form	References
2,2,6,6-tetramethylpiperidine-1-loxy (TEMPO)-oxidized nanocellulose	Hydrogel	[27]
Hyaluronic acid (HA)/ carboxymethyl cellulose (CMC)	Hydrogel	[28]
HA/CMC/Poly (D, L-lactide-co-glycolide) (PLGA)	Hydrogel	[29]
furfuryl hyaluronic acid	Film	[30]
Carboxymethyl chitosan (CMChi), CMC, collagen	Film	[31]
Chitosan (Chi)	Hydrogel	[32]
N, O-carboxymethyl chitosan (N, O-Chi)/oxidized regenerated cellulose (ORC)	Film	[33]
Polyethylene glycol (PEG)/Polylactic acid (PLA)	Film	[34]
silk fibroin protein (SFP)/ Polyvinyl alcohol (PVA), SFP/PEG, SFP/ polyethylene oxide (PEO)	Film	[35]
poly(anhydride-esters)/PEG	Hydrogel	[36]
poly (lactic-co-glycolic acid)-graft-polyvinylpyrrolidone/polyiodide (PLGA-g-PVP/I)	Film	[37]
PLGA/ poly(lactide-co-caprolactone) (PLCA)/poly (L-phenylalanine-co-p-dioxanone (PDPA)	Film	[38]
polypropylene (PP)/poly ε-caprolactone (PCL)/ ORC	Film	[39]
R-CPC copolymer (PCL−polypropylene glycol (PPG)−PEG−PPG−PCL)	Hydrogel	[40]
PVA	Hydrogel	[41]
poly(p-dioxanone-co-l-phenylalanine) (PDPA)	Film	[42]

**Table 2:** Various forms of anti-adhesion products.

Biomaterial	Product Name	Form	References
Oxidized regenerated cellulose (ORC)	Surgicel^®^	Film	[45]
ORC	Interceed^®^	Film	[46]
Carboxymethyl cellulose (CMC)	Seprafilm^®^	Film	[47]
Hyaluronic acid (HA)/CMC	Sepragel^®^	Solution	[48]
HA/CMC	Guardix-sol^®^	Hydrogel	[49]
HA/CMC	Sepraspray^TM^	Powder	[50]
HA derivate	Incert	Film	[51]
HA	Hyalobarrier^®^	Hydrogel	[52]
HA	Sepracoat	Solution	[53]
HA derivate	ACP gel	Solution	[54]
Ferric HA	Lubricoat	Solution	[55]
Ferric HA	Intergel^®^	Solution	[56]
HA derivate	Carbylan-SX	Film/spray	[57]
Icodextrin	Adept^®^	Solution	[58]
Dextran	Hyskon^®^	Hydrogel	[59]
Collagen	COVA+^TM^	Hydrogel	[60]
Polyethylene glycol (PEG)	SprayShield^TM^	Spray	[61]
PEG	SprayGel^TM^	Spray	[62]
PEG	Coseal^®^	Hydrogel	[63]
PEG/CMC	Oxiplex^®^	Hydrogel	[64]
Polylactic acid (PLA)-PEG	REPEL-CV	Film	[65]
PLA	SurgiWrap^®^	Film	[66]
Polyvinyl alcohol (PVA)/CMC	A-part Gel^®^	Hydrogel	[67]
Poloxamer/alginate	Guardix-SG	Hydrogel	[68]
Expanded polytetrafluoroethylene (e-PTFE)	Gore^®^Preclude^®^	Film	[69]

## Abbreviation

Ala	Alanine
CMC	Carboxymethyl cellulose
Gly	Glycine
GTA	Glutaraldehyde
IL	Interleukin
HA	Hyaluronic acid
Hyp	Hydroxyprolyl
NO	Nitric oxide
NSAID	Nonsteroidal anti-inflammatory drug
PCL	Poly ε-caprolactone
PEG	Polyethylene glycol
PEO	Polyethylene oxide
PGA	Polyglycolic acid
PGs	Prostaglandin
PLA	Polylactic acid
PVA	Polyvinyl alcohol
POE	Polyoxymethylene
PPG	polypropylene glycol
Ser	Sericine
TNF	Tumor necrosis factor
tPA	Tissue plasminogen activator
